# Supplementary material for: Associations of area-level deprivation with adverse obstetric and perinatal outcomes in Bavaria, Germany: Results from a cross-sectional study
Source: PLoS One. 2020 Jul 20;15(7):e0236020. doi: 10.1371/journal.pone.0236020 (PMC7371156; doi:10.1371/journal.pone.0236020)
Supplement: S1 Table — Odds ratios [95% confidence intervals] of stillbirth rates by Bavarian Index of Multiple Deprivation (BIMD) quintiles after exclusion of one major birth clinic with unusually high rates of stillbirths, unadjusted and adjusted for offspring’s sex, multiple delivery, maternal age > 35 years, diabetes during pregnancy, maternal overweight, excessive gestational weight gain, migration background, single mother status, parity, maternal smoking during pregnancy, substandard use of antenatal care, living in a city (>100,000 inhabitants) and year of birth. Significant associations (p<0.05) are shown in boldface. (DOCX) [file pone.0236020.s001.docx]

**Supplementary table 1.** Odds ratios [95% confidence intervals] of stillbirth rates by Bavarian Index of Multiple Deprivation (BIMD) quintiles after exclusion of one major birth clinic with unusually high rates of stillbirths, unadjusted and adjusted for offspring’s sex, multiple delivery, maternal age > 35 years, diabetes during pregnancy, maternal overweight, excessive gestational weight gain, migration background, single mother status, parity, maternal smoking during pregnancy, substandard use of antenatal care, living in a city (>100,000 inhabitants) and year of birth. Significant associations (p<0.05) are shown in boldface.

| **BIMD** | **Stillbirth, crude** | **Stillbirth, adjusted** |
| --- | --- | --- |
| Quintile 1 (least deprived) | Reference | Reference |
| Quintile 2 | 1.01 [0.90, 1.13] | 0.89 [0.77, 1.03] |
| Quintile 3 | 0.90 [0.78, 1.03] | **0.84 [0.72, 0.99]** |
| Quintile 4 | 0.95 [0.83, 1.09] | 0.86 [0.73, 1.01] |
| Quintile 5 | 1.06 [0.93, 1.21] | 0.89 [0.76, 1.05] |
| Overall trend (linear increase per quintile) | 1.00 [0.98, 1.03] | 0.97 [0.94, 1.01] |
