# Supplementary material for: Associations of area-level deprivation with adverse obstetric and perinatal outcomes in Bavaria, Germany: Results from a cross-sectional study
Source: PLoS One. 2020 Jul 20;15(7):e0236020. doi: 10.1371/journal.pone.0236020 (PMC7371156; doi:10.1371/journal.pone.0236020)
Supplement: S2 Table — Odds ratios [95% confidence intervals] for primary (n = 148,225 (17.9%) observations) and secondary CS (n = 131,731 (15.9%) observations) by Bavarian Index of Multiple Deprivation (BIMD) quintiles, unadjusted and adjusted for offspring’s sex, multiple delivery, maternal age > 35 years, diabetes during pregnancy, maternal overweight, excessive gestational weight gain, migration background, single mother status, parity, maternal smoking during pregnancy, substandard use of antenatal care, living in a city (>100,000 inhabitants) and year of birth. Significant associations (p<0.05) are shown in boldface. (DOCX) [file pone.0236020.s002.docx]

**S2 Table.** **Associations with primary and secondary caesareans sections (CS).** Odds ratios [95% confidence intervals] for primary (n=148,225 (17.9 %) observations) and secondary CS (n=131,731 (15.9 %) observations) by Bavarian Index of Multiple Deprivation (BIMD) quintiles, unadjusted and adjusted for offspring’s sex, multiple delivery, maternal age > 35 years, diabetes during pregnancy, maternal overweight, excessive gestational weight gain, migration background, single mother status, parity, maternal smoking during pregnancy, substandard use of antenatal care, living in a city (>100,000 inhabitants) and year of birth. Significant associations (p<0.05) are shown in boldface.

| **BIMD** | **Primary CS, crude** | **Primary CS, adjusted** | **Secondary CS, crude** | **Secondary CS, adjusted** |
| --- | --- | --- | --- | --- |
| Quintile 1 (least deprived) | Reference | Reference | Reference | Reference |
| Quintile 2 | **0.93 [0.92, 0.94]** | **1.03 [1.01, 1.05]** | **0.98 [0.97, 0.998]** | **0.91 [0.89, 0.93]** |
| Quintile 3 | **1.05 [1.03, 1.07]** | **1.10 [1.07, 1.12]** | **0.95 [0.93, 0.97]** | **0.98 [0.95, 0.996]** |
| Quintile 4 | **1.05 [1.03, 1.07]** | **1.05 [1.03, 1.07]** | **0.82 [0.80, 0.84]** | **0.79 [0.77, 0.81]** |
| Quintile 5 | **0.96 [0.95, 0.98]** | **1.11 [1.09, 1.14]** | **0.80 [0.79, 0.82]** | **0.77 [0.75, 0.79]** |
| Overall trend (linear increase per quintile) | **1.01 [1.003, 1.01]** | **1.02 [1.02, 1.03]** | **0.94 [0.94, 0.95]** | **0.94 [0.94, 0.95]** |
