# Supplementary material for: Associations of area-level deprivation with adverse obstetric and perinatal outcomes in Bavaria, Germany: Results from a cross-sectional study
Source: PLoS One. 2020 Jul 20;15(7):e0236020. doi: 10.1371/journal.pone.0236020 (PMC7371156; doi:10.1371/journal.pone.0236020)
Supplement: Attachment — Submitted filename: ResponseLetter.docx [file pone.0236020.s003.docx]

Dear Dr. Navaneetham,

thank you for considering our paper for publication in *PLOS ONE.* We appreciate the constructive comments of the reviewers and have changed the manuscript according to their suggestions, using highlighting mode. We have answered the comments as follows:

**Journal Requirements:**

**When submitting your revision, we need you to address these additional requirements:**

**1. Please ensure that your manuscript meets PLOS ONE's style requirements, including those for file naming. The PLOS ONE style templates can be found at** [**http://www.plosone.org/attachments/PLOSOne_formatting_sample_main_body.pdf**](http://www.plosone.org/attachments/PLOSOne_formatting_sample_main_body.pdf) **and** [**http://www.plosone.org/attachments/PLOSOne_formatting_sample_title_authors_affiliations.pdf**](http://www.plosone.org/attachments/PLOSOne_formatting_sample_title_authors_affiliations.pdf)

Thank you for pointing this out to us. We have adjusted the manuscript layout accordingly.

**2. Thank you for submitting the above manuscript to PLOS ONE. During our internal evaluation of the manuscript, we found significant text overlap between your submission (mainly within the discussion section) and the following previously published work, on which you are an author.**

[**https://bmcpregnancychildbirth.biomedcentral.com/articles/10.1186/s12884-016-1060-3**](https://bmcpregnancychildbirth.biomedcentral.com/articles/10.1186/s12884-016-1060-3)

**We would like to make you aware that copying extracts from previous publications, especially outside the methods section, word-for-word is unacceptable. In addition, the reproduction of text from published reports has implications for the copyright that may apply to the publications.**

**Please revise the manuscript to rephrase the duplicated text, cite your sources, and provide details as to how the current manuscript advances on previous work. Please note that further consideration is dependent on the submission of a manuscript that addresses these concerns about the overlap in text with published work.**

**We will carefully review your manuscript upon resubmission, so please ensure that your revision is thorough.**

Thank you for this hint. We have revised the text accordingly.

**3. We note that you have indicated that data from this study are available upon request. PLOS only allows data to be available upon request if there are legal or ethical restrictions on sharing data publicly. For information on unacceptable data access restrictions, please see** [**http://journals.plos.org/plosone/s/data-availability#loc-unacceptable-data-access-restrictions**](http://journals.plos.org/plosone/s/data-availability#loc-unacceptable-data-access-restrictions)**.**

**In your revised cover letter, please address the following prompts:**

**a) If there are ethical or legal restrictions on sharing a de-identified data set, please explain them in detail (e.g., data contain potentially identifying or sensitive patient information) and who has imposed them (e.g., an ethics committee). Please also provide contact information for a data access committee, ethics committee, or other institutional body to which data requests may be sent.**

**b) If there are no restrictions, please upload the minimal anonymized data set necessary to replicate your study findings as either Supporting Information files or to a stable, public repository and provide us with the relevant URLs, DOIs, or accession numbers. Please see** [**http://www.bmj.com/content/340/bmj.c181.long**](http://www.bmj.com/content/340/bmj.c181.long) **for guidelines on how to de-identify and prepare clinical data for publication. For a list of acceptable repositories, please see** [**http://journals.plos.org/plosone/s/data-availability#loc-recommended-repositories**](http://journals.plos.org/plosone/s/data-availability#loc-recommended-repositories)**.**

**We will update your Data Availability statement on your behalf to reflect the information you provide.**

In order to make de-identified data available for public use prior permission must be obtained from the Bavarian institute for quality assurance (‘BAQ’, Prof. Dr. Hermanek, [mail@baq-bayern.de](mailto:mail@baq-bayern.de)). The reason is that the data is expressly gathered for purposes of quality improvement of obstetrics and neonatology care. Hence, the BAQ steering committee must assert that any request for third party usage of the data is in line with the general BAQ objectives. The BAQ is bound by corresponding contracts with its participating hospitals to guarantee adherence to this protocol. The analysis code including all relevant dataset and variable names is available at https://osf.io/mc3te/.

**4. We note that you have included the phrase “data not shown” in your manuscript. Unfortunately, this does not meet our data sharing requirements. PLOS does not permit references to inaccessible data. We require that authors provide all relevant data within the paper, Supporting Information files, or in an acceptable, public repository. Please add a citation to support this phrase or upload the data that corresponds with these findings to a stable repository (such as Figshare or Dryad) and provide and URLs, DOIs, or accession numbers that may be used to access these data. Or, if the data are not a core part of the research being presented in your study, we ask that you remove the phrase that refers to these data.**

Thank you for pointing this out. We have replaced „data not shown“ by specific odds ratios now.

**5. Please include captions for your Supporting Information files at the end of your manuscript, and update any in-text citations to match accordingly. Please see our Supporting Information guidelines for more information:** [**http://journals.plos.org/plosone/s/supporting-information**](about:blank)**.**

Thank you very much, we have changed this accordingly.

**Review Comments to the Author

Reviewer #1: Associations of area-level deprivation with adverse obstetric and perinatal outcomes in Bavaria, Germany: Results from a cross-sectional study is a well written, important and interesting manuscript. The aim of the study is clear and the conclusions are well fit to their findings and any limitations were also taken into account.**

**My main point of criticism is that the description of the data used in the analyses is very limited and used definitions are not always clear.

lines 63-65: neighborhood deprivation indices not only serve as an alternative when individual level SES metrics are not available, but also capture other aspects of deprivation, which are often missed when only looked at individual level metrics. It also captures local availability of care and other area specific care and policy structures. The authors should elaborate more on these assets of area measures**

We thank the reviewer for these important indications and have changed the text accordingly:

“Area-level deprivation research may consider the proportion of deprived people in an area (‘compositional meaning’), the presence of possible area effects beyond the local concentration of deprived people (’collective meaning’), and the lack of infrastructural facilities or other area features (‘environmental meaning’) [15]. Area-level deprivation is often measured with standardised composite deprivation indices describing a structural lack of material and social resources in an area, considering e. g. income and occupation but also municipal/district revenues and environmental indicators. Therefore, these indices are valuable instruments for the analysis of area effects on health beyond the individual level, even when they might be used as a surrogate when individual socioeconomic data are not available [16].” **line 67: introduce abbreviation BIMD here instead of in method section**

Thank you very much, we have changed this accordingly.

**How is the BIMD calculated? on a multiplicative or additive scale? is it standardized? is this only available for Bavaria? or also for the rest of Germany (and how does the level of deprivation in Bavaria compare to Germany)?**

Thank you very much, we acknowledge the need to integrate more information on the BIMD in the text and have added following information in the background section:

“Based on an established British method [15], the BIMD is a multidimensional construct combining standardised and transformed indicators in specific deprivation domains which are weighted and combined in an overall deprivation index. The BIMD was originally developed as a model for the development of the German Index of Multiple Deprivation (GIMD), being today part of a number of Indices of Multiple Deprivation (IMD) for Germany and some of its federal states. More on the methodology of the German IMD, the area-level distribution of deprivation in Germany and its association with health can be found elsewhere [17-20].”

**How do the individual domains relate to the composite? and why only look for a linear trend over the domains and not like the main analyses (line 98)?**

The individual domains load with a certain amount on the composite score. We have added these amounts (in %) to the main text. We decided to investigate them with a linear trend only in order to reduce multiple testing issues.

**line 87: what is the size (plus range) of a district?**

By the reporting date of 31 December 2016, the smallest district in Bavaria had a population of 40.7 thousand inhabitants (urban district of Schwabach), whereas the largest district (city of Munich) had a population of 1.46 million inhabitants (range: 1.42 million). The median number of inhabitants was 115.6 thousand. We have now inserted additional information on the size of the districts in the text.

**line 91: "We used data on all deliveries..." where there any restrictions to be included in the dataset? like minimal amount of weeks of gestation of minimal birth weight? Viability? This has considerable implications for the interpretation of your findings.**

No, we deliberately applied no inclusion restrictions on our data.

**How were the stillbirths defined?**

All neonates without signs of life and minimum birth weight of 500 g where counted as stillbirths.

**Was there a distinction possible between primary and secondary caesareans? This distinction should be made since their role in the management of deliveries is very different. The associations with deprivation of both types could well be different and capture different aspects of inequality in care provision**

Thank you for this interesting suggestion. We added a supplementary analysis for primary and secondary sectio (S2 Table) and commented on it in the main text.

**lines 97-98: which analyses do you refer to here? The trend over the quintiles? It is not entirely clear to me which results match these analyses**

Yes, this refers to the ‚overall trend‘ analyses. We have added this term for clarity. **why did you choose to correct for multiple pregnancies instead of stratifying? Were the number of multiple pregnancies comparable over deprivation quintiles? most studies limit their analyses to singletons only and now it is more difficult to directly compare your results with that of others.**

There were two reasons why we did not stratify for multiple pregnancies: First, this would have doubled the number of analyses and hence contributed to multiple testing issues. Second, the statistical power in the subset of multiple pregnancies would likely have been too low to draw valid conclusions. **did you have information on other birth modes than CS only? if yes, why not correct for that?**

Thank you. The only other birth mode available in addition to vaginal birth and CS was vaginal surgery. We added this variable to the sensitivity analysis for Apgar scores. **how did you handle missing data? how was missing covariate data distributed among the deprivation quintiles and outcomes?**

We added the following to the Results and Discussion, respectively:

„Missing covariate data occurred for n=28,419 observations (13.9 %) in Q1, n=45,553 (17.7 %) in Q2, n=23,108 (19.4 %) in Q3, n=24,634 (21.2 %) in Q4 and n=26,301 (20.2 %) in Q5, respectively. In pregnancies with missing covariate data, slightly higher prevalences were observed for all outcomes (preterm delivery: 9.1 %, stillbirth: 0.4 %, CS: 35.9 %, SGA: 9.6 %, low Apgar score: 1.3 %).“

„Missing covariate data were not evenly distributed across BIMD quintiles and pregnancy outcomes. We therefore cannot preclude that our adjusted analyses were affected by attrition bias, although again we do not think this is a major issue here because the differences in the proportions of missing covariate data were not very large, and the results from the unadjusted and adjusted analyses were quite similar in most cases.“

**were there specific rules in place to define unusually high rates of stillbirths? or was it a post-hoc decision to see whether the outlier clinic influenced your findings? If so, than please state that... the current sentence is very vague about this point/decision**

Thank you. We have added that this was a posthoc decision. **I would like to see a table displaying the prevalence of each outcome, for the whole population and over the quintiles**

Thank you for this suggestion. We have added this table as new table 2.

**Tables 2-4: please provide additional information about the N for analyses, per outcome and per quintile. This is especially of interest since 'single mother' is a covariate with a large amount of missing data, and this covariate is likely not evenly distributed among the quintiles, potentially introducing bias**

This issue has already been addressed by our answer to your other question related to missing covariates further above: As there were no missing values for any of the outcome variables, the N per quintile was the same for each adjusted analysis, irrespectively of the outcome variable.

**lines 129-136: this paragraph on the different domains was hard to read, not sue what the message here is**

Thank you. We have revised this paragraph and hope that it reads clearer now.

**line 138: please provide ORs + 95%CIs for these analyses instead of "data not shown"**

Thank you. We followed your advice.

**line 139: what was the size of the excluded clinic? how many births were excluded from the analyses?**

Due to data protection reasons, we cannot mention the exact size of this clinic. We added the information that this clinic had >20,000 births in 2009-2016. **lines 151-153: this may be influenced by different definitions; maybe the increased amount of perinatal death in other studies focus on the period just after birth, while yours could just focus on IFD? or other mechanisms... Also, what were the unadjusted estimates for this outcome? do they differ? on the other hand, your amount of stillbirths is low, which could influence your findings. also, selective reporting of stillbirths in the database could have played a role in this. Please take this into account in discussing your findings on stillbirths in particular.**

Thank you for addressing this issue. The unadjusted and adjusted ORs for stillbirths were relatively similar, as shown in table 3. Selective reporting of stillbirths is unlikely, because stillbirths are a key outcome for benchmarking of birth clinics and is therefore rigorously checked during data collection, as was already mentioned in the discussion.

**lines 159-161: were the apgar scores (but also SGA and preterm birth) only counted for live births or any births? This could have influenced the found associations (similar to healthy survivor bias) and should be addressed**

All outcomes were counted for any births. We added one sentence about this to the Discussion.

**lines 170-172: similar to my suggestion in the background; area SES is not just an 'alternative' to use when individual level SES is absent, it captures other aspects of SES and also is also associated with health outcomes, irrespective of individual level SES (like in the study of Daoud BMC Pregnancy Childbirth. 2015)**

Thank you. We have removed the respective sentence.

**I miss the limitation that it was a cross-sectional study; especially since there is ample evidence suggestion that SES is not a fixed thing, but evolves over time and the effect of SES differs with different degrees and lengths of exposure.**

Thank you. We have added the following sentence: „The fact that our analyses were based on cross-sectional data constitutes a limitation, as we were not able to investigate potential changes in deprivation over time and their impact on pregnancy outcomes.“

We hope that all objections of the Reviewer could be sufficiently addressed and would be pleased if the amended manuscript would be accepted for publication.

Yours sincerely,

Andreas Beyerlein (for all authors)
