# Supplementary material for: Associations of area-level deprivation with adverse obstetric and perinatal outcomes in Bavaria, Germany: Results from a cross-sectional study
Source: PLoS One. 2020 Jul 20;15(7):e0236020. doi: 10.1371/journal.pone.0236020 (PMC7371156; doi:10.1371/journal.pone.0236020)
Supplement: Attachment — Submitted filename: ResponseLetter2.docx [file pone.0236020.s004.docx]

Dear Dr. Navaneetham,

thank you for considering our paper for publication in *PLOS ONE.* We have answered the Reviewer’s remaining comments as follows:

**Reviewer #1: The vast majority of the comments/concerns raised have been successfully covered by the authors.
I have two minor but important points that should be changed in the manuscript.**

**1. definition of stillbirths: please include the used definition in the methods section. Also, you described in the rebuttal, that all neonates without signs of life and minimum birth weight of 500 g where counted as stillbirths. but since you have no restrictions for the population used in the analyses, how do you deal with any birth with a birth weight below 500gr? if they are excluded, than please add that to the description of your population, if they were dealt with in another way, plase describe this in the ethods section**

Thank you for raising this issue. Stillborn neonates below a birth weight of 500 g are not required for official registration in Germany and hence they are also not part of the database. This means that neonates with a birth weight below 500 g enter the database only if they show signs of life, but not if they are stillbirths. We have clarified this in the main text.

**2. internationally, the term primary CS is use dto describe the elective or planned CS, and secundary CS those following complications when labour had already started. in your discussion section you use the terms the other way around. please correct this.** 

The distinction between primary and secondary Caesarean sections is in fact - contrary to your assumption – a German rather than an International Convention. Internationally the distinction is generally between elective and emergency Caesarean sections. However, when referring to primary and secondary Caesarean sections as we did in our article we clearly intend to use the term primary for such Caesarean sections when conducted prior to onset of Labour as distinguished from so called secondary Caesarean sections which are defined as Caesarean sections conducted after the onset of Labour. We apologize for the confusion and have revised this part of the Discussion accordingly.

We hope that all objections of the Reviewer could be sufficiently addressed and would be pleased if the amended manuscript would be accepted for publication.

Yours sincerely,

Andreas Beyerlein (for all authors)
